# Supplementary material for: G-quadruplex RNA motifs influence gene expression in the malaria parasite Plasmodium falciparum
Source: Nucleic Acids Res. 2021 Nov 18;49(21):12486–501. doi: 10.1093/nar/gkab1095 (PMC8643661; doi:10.1093/nar/gkab1095)
Supplement: gkab1095_Supplemental_Files [file gkab1095_supplemental_files.zip › Merged Supp figs and legends.pdf]

G-quadruplex RNA motifs influence gene expression in the malaria parasite *Plasmodium falciparum*

SUPPLEMENTARY FILES

Supp figure 1: **QUMA-1 staining of *P. falciparum* parasites**

Parasites were removed from their host cell and fixed in formaldehyde. Enzymatic treatments to selectively remove RNA or DNA were performed, before staining with QUMA-1 and DAPI.

Supp figure 2: **Examples of canonical and non-canonical rG4 structures**

(A) Planar structure of a G-quartet.

(B) Representative structure of a canonical rG4 with a G_3N_{1-7} sequence consensus, which is composed of three layers of G-quartet stabilized by potassium ions (K^+).

(C, D) Representative structures of (C) non-canonical rG4s and (D) variant non-canonical rG4s described in this study. These rG4s are classified by the properties of their structural imperfections relative to the canonical rG4.

Supp figure 3: **Biophysical analysis of the non-canonical rG4s found in rG4-forming sequence**

(A-D) CD assay on non-canonical rG4s, (A) PF3D7_0420300_rG4, (B) PF3D7_0525400_rG4, (C) PF3D7_0724900_rG4, and (D) PF3D7_1031200_rG4. All four oligos show a negative peak at ~240 nm and positive peak at ~262 nm, with a higher signal in K+ condition compared to Li+ condition. This suggests the formation of a parallel rG4 conformation.

(E-H) UV melting assay on the same rG4s in (A-D). The observed Tm are in the range of 50-70 ˚C, indicating the formation of thermostable rG4s in physiological conditions.

(I-J) Ligand enhanced fluorescence spectra showing the excitation peaks on the same oligos in (A-D). (I) Under NMM conditions, the fold difference average for all four oligos are 1.4, 7.7, 4.4 and 4.7, respectively. (J) Under ISCH-oa1 conditions, the fold difference average for all four oligos are 2.1, 11.3, 4.2 and 4.0, respectively. The fluorescence intensities of all oligos are higher in K+ condition than Li+ condition, suggesting the rG4 structure formation.

Supp figure 4: **Functional associations of rG4-harbouring protein-coding genes**

(A) Results of GO 'biological process' enrichment analysis on genes that harbour rG4s belonging to the 4 canonical/non-canonical rG4 structural motifs. Only enriched GO terms with a p value < 0.05 and FDR < 0.05 are shown.

(B) Summary of the most common GO biological processes among genes that harbour rG4s belonging to the 4 canonical/non-canonical rG4 structural motifs. Three groups of biologically-related GO terms are highlighted, with examples of corresponding rG4-harbouring genes.

Supp figure 5: **Biophysical analysis of the rG4-forming sequence from the AP2 reporter gene**

(A, B) Ligand enhanced fluorescence spectra showing excitation peak of rG4-encoding oligos (wild, wildtype sequence; Mut, rG4-mutated sequence) folded under K+ as compared to Li+ conditions in the presence of rG4 ligands NMM (A) and ISCH-oa1 (B). For NMM, the fold difference average is 14.6 for wild type rG4 and 2.3 for mutant rG4. For ISCH-oa1, the fold difference average is 7.9 for wild type rG4 and 4.1 for mutant rG4.

(C, D) Circular dichroism performed on the same oligos in (A, B). Blue and red lines represent folding in K+ and Li+ ions. (C) CD spectrum of wild type sequence, showing a negative peak at ~240 nm and positive peak at ~262 nm, suggesting the formation of parallel topology. (D) CD spectrum of mutant sequence showing a lesser sign of rG4 formation in both K+ and Li+ conditions.

(E-G) UV Melting performed on the same oligos in (A, B). (E) Concentration dependent UV melting of wild type oligo in K+ condition, (F) concentration dependent UV melting of mutant oligo in Li+ condition, (G) a different representative UV melting of wild type oligo in K+ condition with both forward and reverse pathway. The observed Tm of the wild type is higher than the Li+ condition, indicating the physiological stability of the rG4 under K+ conditions.

Supp figure 6: ***In vitro* folding of rG4 motifs from reporter genes**

QUMA-1 fluorescence assays on RNA oligos encoding (A) the rG4 from the *AP2* gene and (B) the equivalent non-rG4 from the *Rifin* gene in the presence of LiCl or KCl. Two versions of each sequence were tested, the WT transcript and the rG4mut transcript where the sequence has been modified to prevent rG4 formation. Ligand fluorescence shows that an rG4 is formed by the WT *AP2* sequence, is largely ablated in the rG4mut sequence, and is absent in LiCl instead of KCl. The *Rifin* sequences form no rG4s, as expected.

Supp figure 7: **Translation efficiency of all rG4-encoding genes, and of selected rG4-encoding reporter genes *AP2* and *Rad54 in vivo* at 4 lifecycle stages**

(A, B) Translation efficiency of all expressed genes in the transcriptome at four stages of the erythrocytic lifecycle, based on data from (37), in the form of either box-and-whisker plots showing mean and interquartile range of Log(TE) (A) or histograms of Log(TE) values (B). Genes with *bona fide* rG4s (detected by rG4-Seq) are in red while genes predicted to be rG4-null are in blue. p-values were calculated using two-tailed Student's T-test: ***, $p<0.001$. Arrows accompanying the p-values show the direction of the shift in TE for rG4-genes.

(C-D) Profiles of Ribo-Seq coverage and mRNA-Seq for two of the selected rG4 reporter genes, *AP2* (C) and *Rad54* (D), shown across lifecycle stages. Transcription of the *AP2* gene peaks in late trophozoites and schizonts; transcription of the Rad54 gene peaks in late trophozoites. Data obtained from [28], visualised via the Mochiview browser. The locations of the rG4s are marked.

Supp Figure 8: **Biophysical characterization of Rad54 rG4 oligonucleotides**

RNA oligos encoding Rad54 WT, rG4mut, the common field mutation (G-to-T) and the uncommon mutation (G-to-A) were tested in two complementary biophysical assays.

(A-D) CD spectrum of each oligo at room temperature under K^+ and Li^+ conditions. The spectra show that the rG4mut and uncommon field sequences do not show the formation of rG4s, while the WT and common field sequences show the formation of an rG4 in parallel topology. (UV melting data shown in Figure 6G further reveal that the uncommon field sequence can in fact form a weak rG4 but this melts at 18°C, below the room temperature used in this assay, whereas the common field sequence forms an rG4 that melts at 30.5°C, above room temperature).

(E-H) NMM and ThT enhanced fluorescence spectroscopy under K^+ and Li^+ conditions. The fluorescence intensities increase as follows: Rad54 G4mut < Rad54 uncommon field < Rad54

common field < Rad54 rG4 WT. This confirms the UV melting data shown in Figure 6G on the relative stabilities of these rG4s. The WT oligo forms a stable rG4 in K^+ and is likely to form an rG4 even under Li^+ conditions, in the presence of stabilizing ligands, whereas all three mutant oligos form weaker rG4s and cannot be induced by stabilizing ligands to fold in Li^+.

Supp Table 1 **Gene-level summary of predicted $(G_3N_x)_4$ PQSs and their detection status in rG4-seq**

Supp Table 2 **Analysis of overlap between DNA G4s detected in G4-seq and RNA G4s detected in rG4-seq**

(A) Distribution of experimentally determined DNA G4s in *P. falciparum* G4-seq, annotated by genomic regions. (B) Statistics on overlaps between G4-seq-detected DNA G4s residing in the sense strand of exons and rG4-seq-detected RNA G4s. (C) Statistics on overlaps between G4-seq DNA G4s residing in the sense strand of exons, CDS regions and low complexity peptide regions (LCRs).

Supp Table 3 **Oligo sequences used in this study**

Supp Table 4 **Statistical analysis of *in vitro* translation data**

Detailed output of the 2-way ANOVA for the results of *in vitro* transcription of *AP2* and *Rif12* genes. A Bonferroni correction for multiple testing was applied. Statistics were obtained using GraphPad Prism.

Supp Table 5 **Genetic variation found in field strains of *P. falciparum* in the rG4-encoding sequence of the *Rad54* gene**

Summary of all SNPs and indels in >10,000 geographically-diverse field strains of *P. falciparum* that could affect the rG4-encoding sequence in the Rad54 gene. Data collected from the MalariaGEN *P. falciparum* Community Project, v6.0, released 13th Nov 2020.

Supp Data File 1 **List of 2,569 *P. falciparum* rG4s detected by rG4-seq**

Supp Data File 2 **Prediction outcome and repeat annotations of 335 $(G_3N_x)_4$ PQSs in *P. falciparum* protein coding genes**

Supp Data File 3 **Overlap between exonic, sense-strand DNA G4s detected in G4-seq and RNA G4s detected in rG4-seq, annotated for coincidence with LCRs**

Supp Figure 1

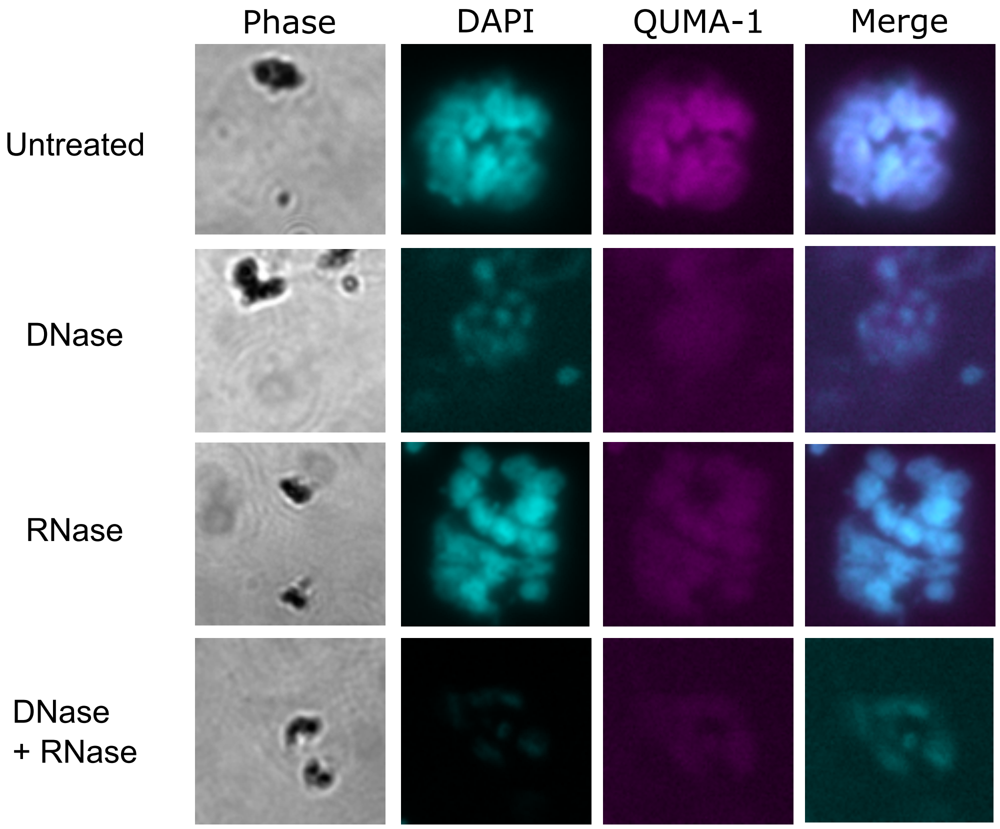

[illegible]

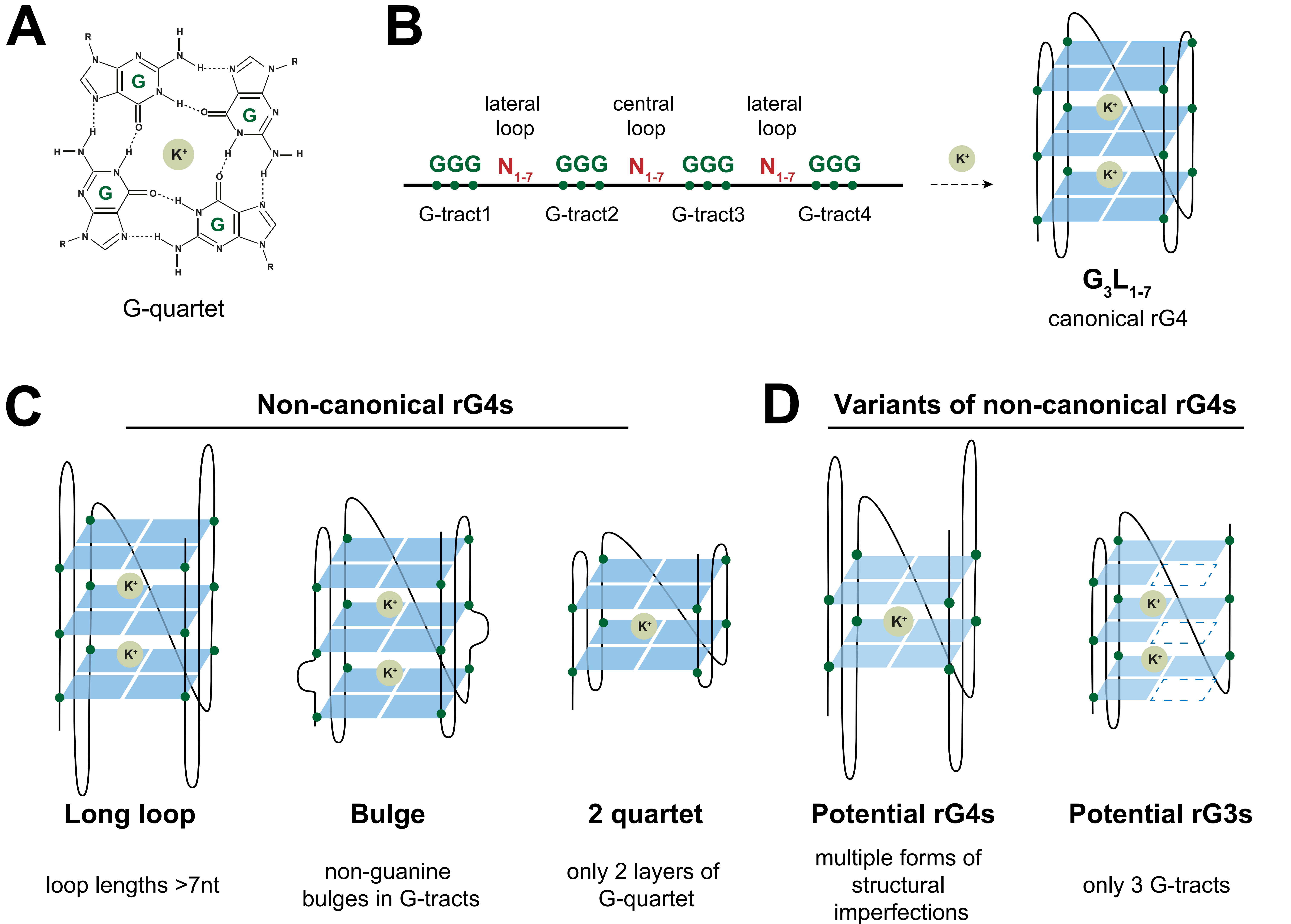

A
G
K+
G-quartet
B
lateral loop
central loop
lateral loop
GGG N1-7 GGG N1-7 GGG N1-7 GGG
G-tract1
G-tract2
G-tract3
G-tract4
K+
G3L1-7
canonical rG4
C
Non-canonical rG4s
Long loop
loop lengths >7nt
Bulge
non-guanine bulges in G-tracts
2 quartet
only 2 layers of G-quartet
D
Variants of non-canonical rG4s
Potential rG4s
multiple forms of structural imperfections
Potential rG3s
only 3 G-tracts

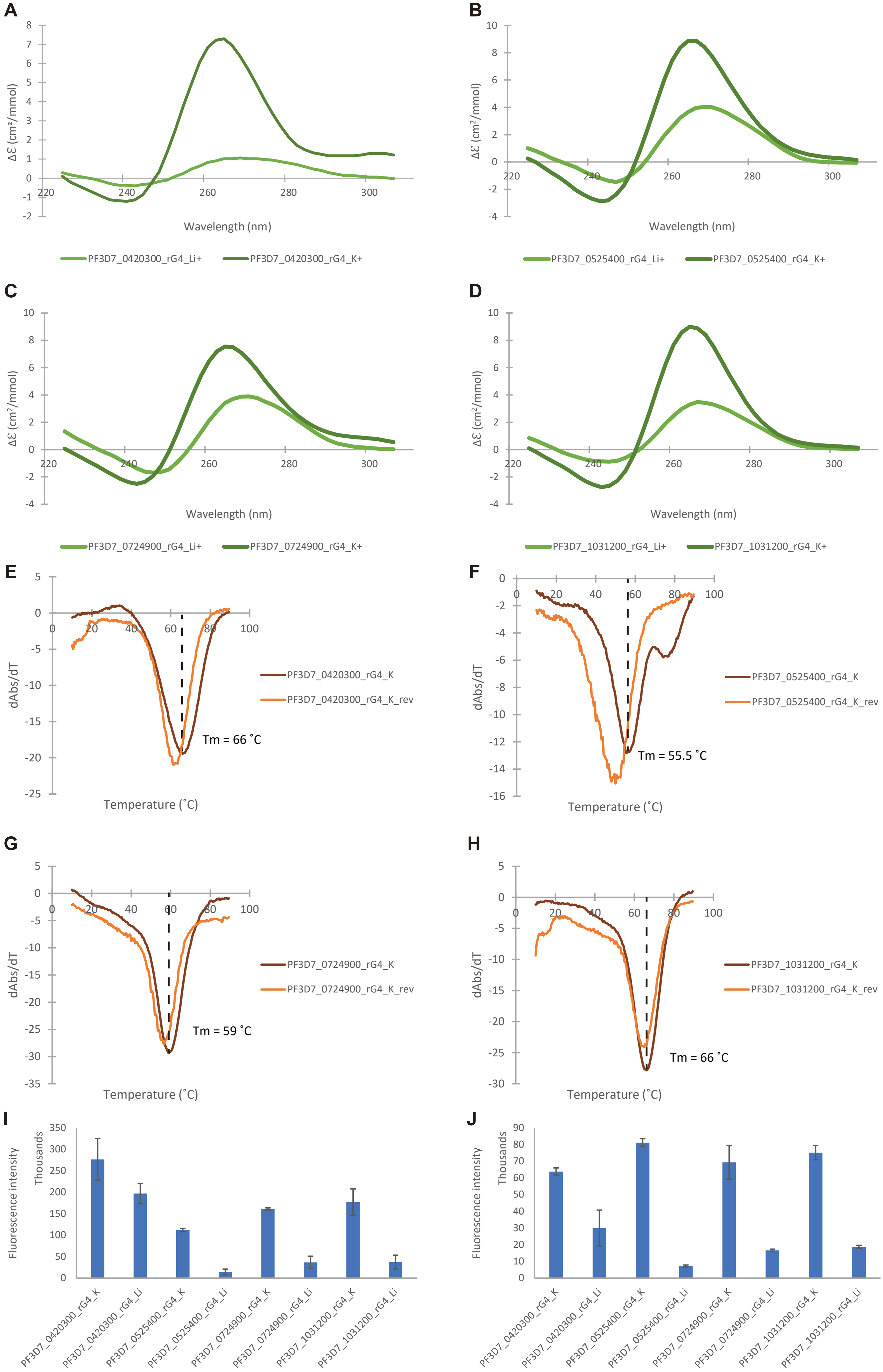

A
ΔƐ (cm²/mmol)
Wavelength (nm)
PF3D7_0420300_rG4_Li+
PF3D7_0420300_rG4_K+
B
ΔƐ (cm²/mmol)
Wavelength (nm)
PF3D7_0525400_rG4_Li+
PF3D7_0525400_rG4_K+
C
ΔƐ (cm²/mmol)
Wavelength (nm)
PF3D7_0724900_rG4_Li+
PF3D7_0724900_rG4_K+
D
ΔƐ (cm²/mmol)
Wavelength (nm)
PF3D7_1031200_rG4_Li+
PF3D7_1031200_rG4_K+
E
dAbs/dT
Temperature (˚C)
Tm = 66 ˚C
PF3D7_0420300_rG4_K
PF3D7_0420300_rG4_K_rev
F
dAbs/dT
Temperature (˚C)
Tm = 55.5 ˚C
PF3D7_0525400_rG4_K
PF3D7_0525400_rG4_K_rev
G
dAbs/dT
Temperature (˚C)
Tm = 59 ˚C
PF3D7_0724900_rG4_K
PF3D7_0724900_rG4_K_rev
H
dAbs/dT
Temperature (˚C)
Tm = 66 ˚C
PF3D7_1031200_rG4_K
PF3D7_1031200_rG4_K_rev
I
Fluorescence intensity
Thousands
PF3D7_0420300_rG4_K
PF3D7_0420300_rG4_Li
PF3D7_0525400_rG4_K
PF3D7_0525400_rG4_Li
PF3D7_0724900_rG4_K
PF3D7_0724900_rG4_Li
PF3D7_1031200_rG4_K
PF3D7_1031200_rG4_Li
J
Fluorescence intensity
Thousands
PF3D7_0420300_rG4_K
PF3D7_0420300_rG4_Li
PF3D7_0525400_rG4_K
PF3D7_0525400_rG4_Li
PF3D7_0724900_rG4_K
PF3D7_0724900_rG4_Li
PF3D7_1031200_rG4_K
PF3D7_1031200_rG4_Li

Supp figure 4

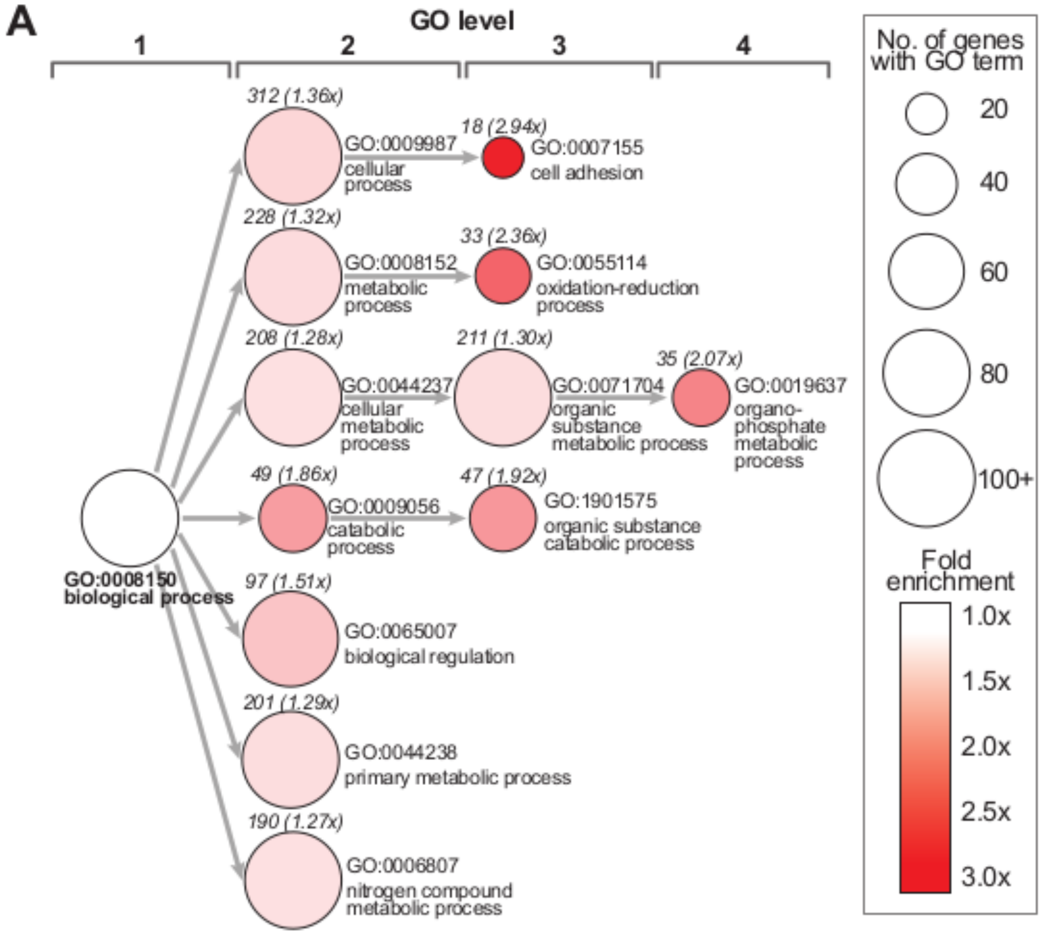

B

Most common GO biological process	No. of genes in P.fa	No. of rG4-harboring genes
pathogenesis	27	20
antigenic variation	22	17
cell-cell adhesion	22	16
modulation by symbiont of host erythrocyte aggregation	21	16
cytoadherence to microvasculature, mediated by symbiont protein	22	16
oxidation-reduction process	33	19
response to drug	50	16
proteolysis	43	10
protein folding	32	9
cell redox homeostasis	14	6
ubiquitin-dependent protein catabolic process	24	5
regulation of transcription, DNA-templated	27	9
translation	133	19
entry into host cell	26	14
metabolic process	77	10
protein phosphorylation	31	10
transport	113	8
protein transport	37	7
transmembrane transport	36	6
tricarboxylic acid cycle	10	6
glycolytic process	11	6
mRNA splicing, via spliceosome	13	5
tRNA aminoacylation for protein translation	18	5
cell cycle	23	5
ribosome biogenesis	8	5

Representative rG4-harboring genes

E3 ubiquitin-protein ligase *(HEUL)*
erythrocyte binding antigen-181 *(EBA-181)*
erythrocyte membrane protein 1 *(PfEMP1)*
Rifin *(RIF)*

calcium-transporting ATPase *(ATP6)*
histone deacetylase 1 *(HDAC1)*
malate:quinone oxidoreductase *(MQO)*
phosphatidylinositol 3-kinase *(PI3K)*
phosphatidylinositol 4-kinase *(PI4K)*

transcriptional coactivator ADA2 *(ADA2)*
AP2 domain transcription factors *(ApiAP2)*
histone acetyltransferase GCN5 *(GCN5)*

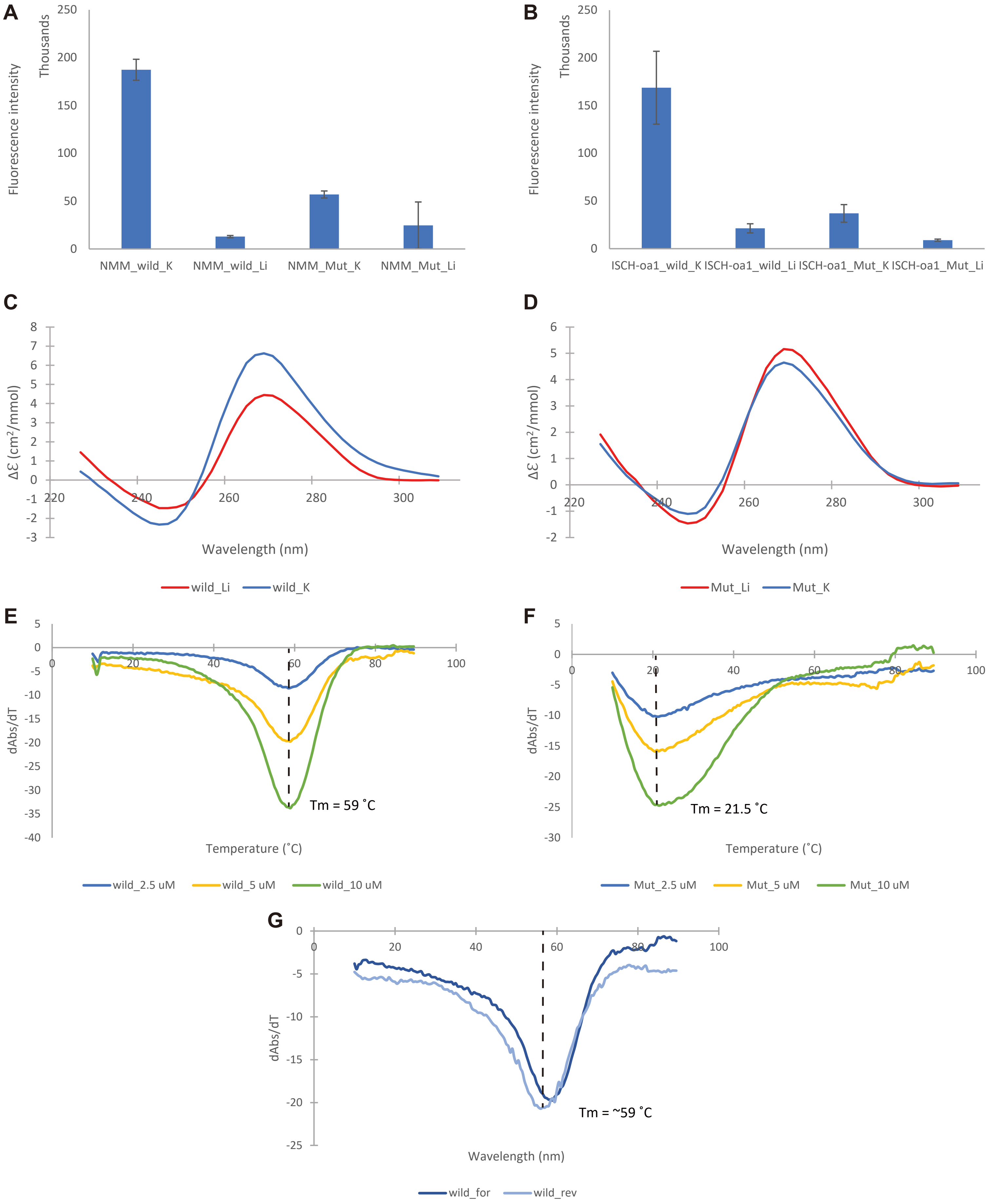

A
Fluorescence intensity
Thousands
250
200
150
100
50
0
NMM_wild_K
NMM_wild_Li
NMM_Mut_K
NMM_Mut_Li
B
Fluorescence intensity
Thousands
ISCH-oa1_wild_K
ISCH-oa1_wild_Li
ISCH-oa1_Mut_K
ISCH-oa1_Mut_Li
C
ΔƐ (cm²/mmol)
Wavelength (nm)
220
240
260
280
300
wild_Li
wild_K
D
ΔƐ (cm²/mmol)
Wavelength (nm)
Mut_Li
Mut_K
E
dAbs/dT
Tm = 59 ˚C
Temperature (˚C)
wild_2.5 uM
wild_5 uM
wild_10 uM
F
dAbs/dT
Tm = 21.5 ˚C
Temperature (˚C)
Mut_2.5 uM
Mut_5 uM
Mut_10 uM
G
dAbs/dT
Tm = ~59 ˚C
Wavelength (nm)
wild_for
wild_rev

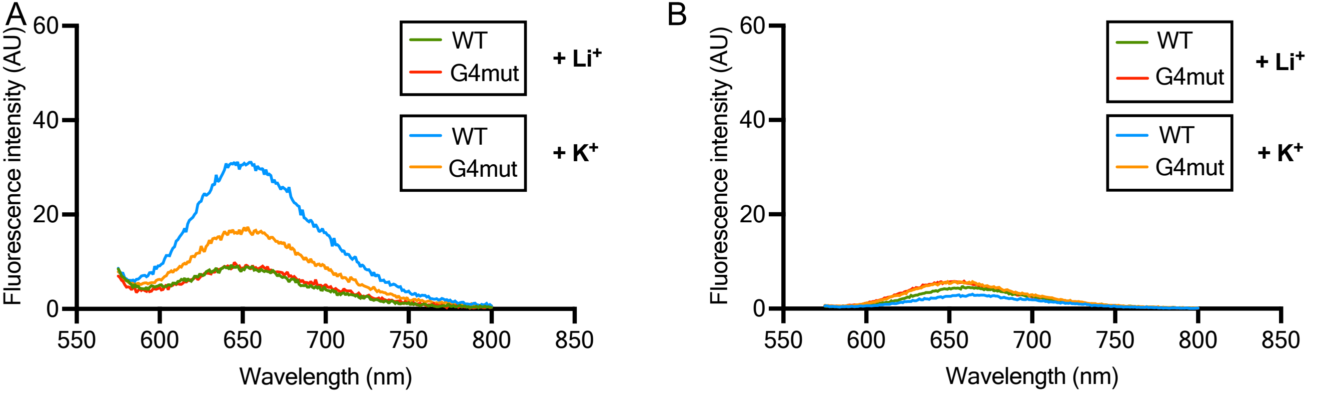
A
B
Fluorescence intensity (AU)
60
40
20
0
550
600
650
700
750
800
850
Wavelength (nm)
WT
G4mut
+ Li+
WT
G4mut
+ K+

Supp Figure 7

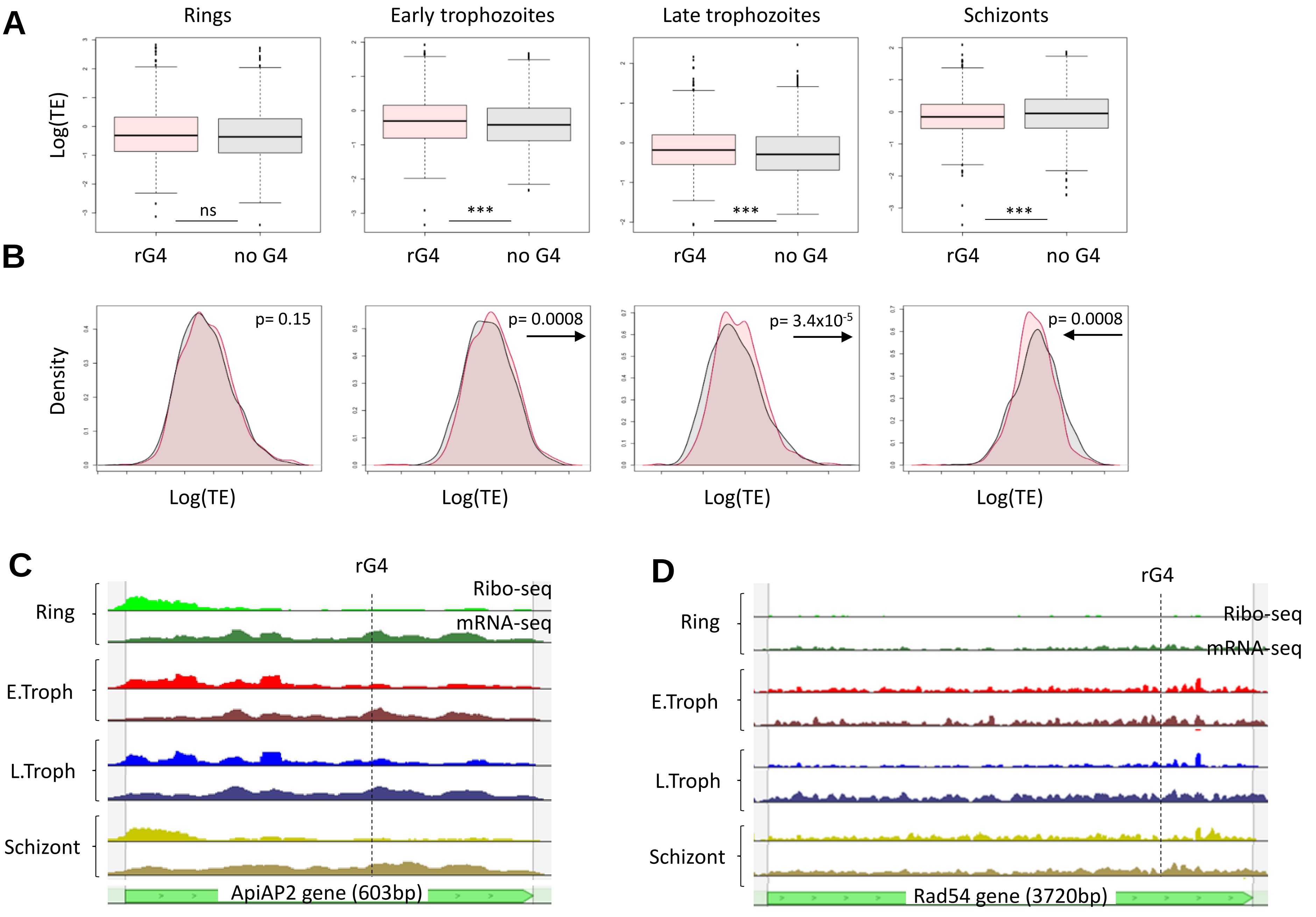

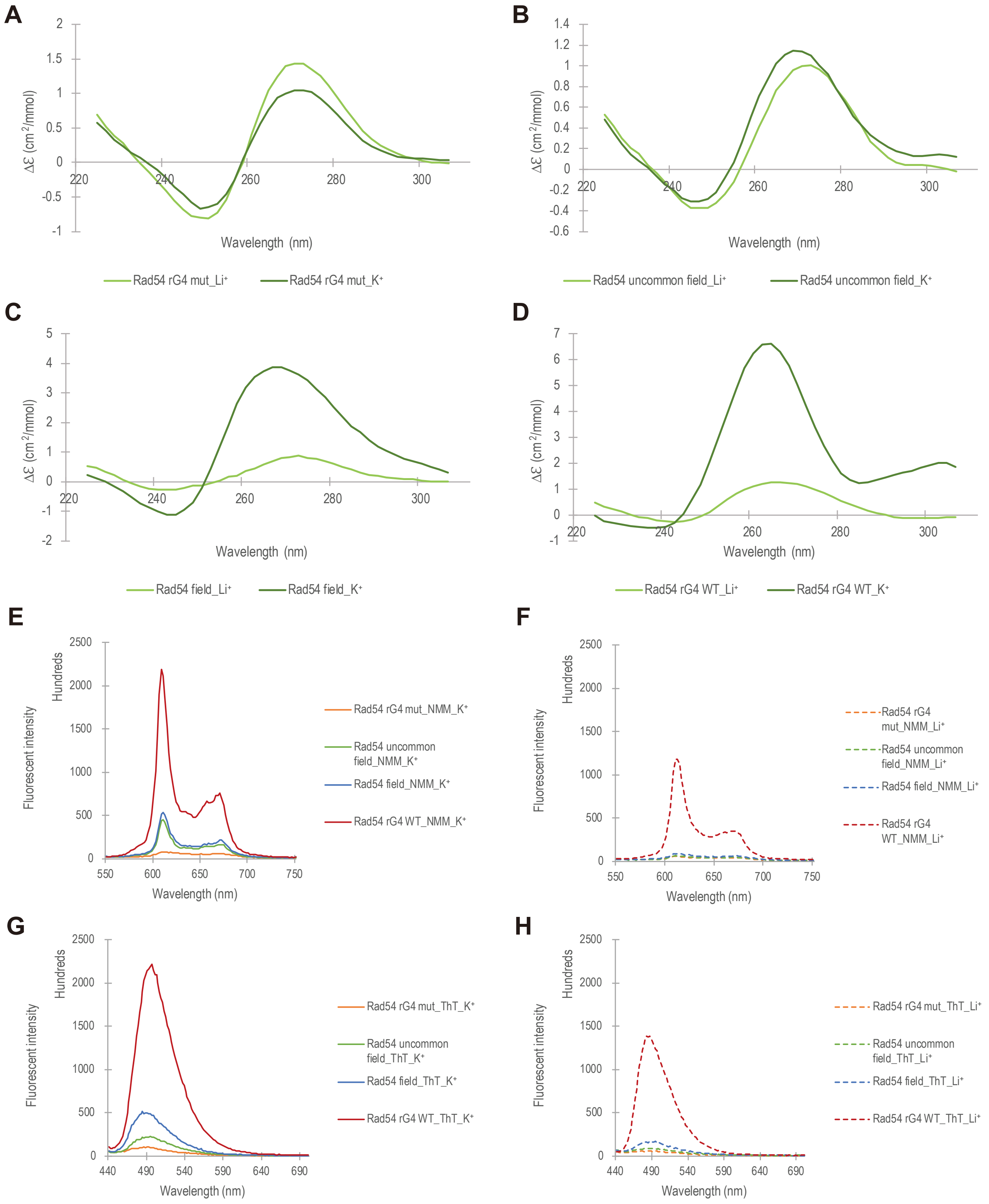
A
ΔE (cm²/mmol)
Wavelength (nm)
Rad54 rG4 mut_Li⁺
Rad54 rG4 mut_K⁺
B
ΔE (cm²/mmol)
Wavelength (nm)
Rad54 uncommon field_Li⁺
Rad54 uncommon field_K⁺
C
ΔE (cm²/mmol)
Wavelength (nm)
Rad54 field_Li⁺
Rad54 field_K⁺
D
ΔE (cm²/mmol)
Wavelength (nm)
Rad54 rG4 WT_Li⁺
Rad54 rG4 WT_K⁺
E
Fluorescent intensity
Hundreds
Wavelength (nm)
Rad54 rG4 mut_NMM_K⁺
Rad54 uncommon field_NMM_K⁺
Rad54 field_NMM_K⁺
Rad54 rG4 WT_NMM_K⁺
F
Fluorescent intensity
Hundreds
Wavelength (nm)
Rad54 rG4 mut_NMM_Li⁺
Rad54 uncommon field_NMM_Li⁺
Rad54 field_NMM_Li⁺
Rad54 rG4 WT_NMM_Li⁺
G
Fluorescent intensity
Hundreds
Wavelength (nm)
Rad54 rG4 mut_ThT_K⁺
Rad54 uncommon field_ThT_K⁺
Rad54 field_ThT_K⁺
Rad54 rG4 WT_ThT_K⁺
H
Fluorescent intensity
Hundreds
Wavelength (nm)
Rad54 rG4 mut_ThT_Li⁺
Rad54 uncommon field_ThT_Li⁺
Rad54 field_ThT_Li⁺
Rad54 rG4 WT_ThT_Li⁺
